# Gastrostomy with peritoneal collar versus percutaneous endoscopic gastrostomy

**Published:** 2016

**Authors:** C Tudor, C Branescu, C Savlovschi, A El-Khatib, H Pantu, A Nica, AM Dascalu, B Masoumeh, AS Tudor, SM Oprescu, D Serban

**Affiliations:** * Upper Digestive Surgery Clinic, University Emergency Hospital, Bucharest, Romania; ** “Carol Davila” University of Medicine and Pharmacy, Bucharest, Romania; *** SC El-Khatib Medica SRL, Bucharest, Romania; **** Medlife Hyper Clinic, Bucharest, Romania

**Keywords:** dysphagia, swallowing difficulties, surgical solution for feeding, percutaneous endoscopic gastrostomy

## Abstract

**Aim.** The present study aimed to perform a medico-surgical comparative analysis of the 2 most widely used techniques: gastrostomy with peritoneal collar versus percutaneous endoscopic gastrostomy, based on the vast clinical experience in an Upper Digestive Surgery Clinic.

**Materials and method.** A retrospective study was carried out between January 2010 and January 2015 on the patients admitted for a surgical solution for feeding. The indications, preoperative preparation, surgical techniques, and postoperative outcomes were analyzed.

**Results.** Out of the 94 cases admitted for a surgical solution for feeding, 67 underwent gastrostomy with peritoneal collar (GPC) and in 27 cases percutaneous endoscopic gastrostomy (PEG) was performed. The indications for GPC were benign or malign causes of dysphagia, the most frequent being malign tumors of tongue, pharynx and larynx (47.76%), advanced inoperable esophageal or eso-cardiac cancers (26,86%), post-caustic esophageal stenosis (10.44%). PEG was performed in patients with functional difficulties of swallowing: sequelae of cerebral vascular accidents (44.44%), low Glasgow Coma Scale Score (29.62%) of different etiologies, Parkinson disease (18.51%) advanced dementia (7.4%), early nasopharyngeal cancer (2 cases). The intraoperatory and postoperatory complications were few and of minor importance in both techniques, but PEG allowed an immediate retake of alimentation (vs. at least 48 hours wait in GPC), with less gastric stasis, biliary reflux and aspiration related respiratory problems.

**Conclusions.** Both techniques are easy and safe to perform, but an appropriate selection is required according to the cause of the swallowing difficulty. In cases with permeable digestive tube, PEG may be an excellent minimally invasive solution, but the costs and availability of the PEG kit and prehydrolyzed nutritive solution, as well as the co-existence of an upper digestive endoscopy service were limitations that had to be taken into account.

## Introduction

As the physiologic way of preparing, processing, and crossing a digestive circuit, feeding has preoccupied the entire health activity both directly or through collateral connections. From swallowing to the absorption of the nutrients, distinct anatomic and physiologic territories are traversed, whose understanding is defining in order to know the vital process. 

As moderator and modeler of the pathophysiology of feeding, the practitioner physician has to face two major categories of problems:

1. Stop - stenosis, the difficulty of crossing through a damaged tube such as an anatomical structure (for any reason);

2. External causes with no anatomical damage of the upper digestive system (which basically functions as an element of contention): reversible or irreversible disorders with a neurological disruption of the central mechanism of swallowing.

In order to solve these 2 main categories of feeding problems, a wide variety of surgical techniques have been described, generally known as surgical solutions of feeding - gastrostomy and jejunostomy. Each of them has as a common goal, the introducing of the alimentary principles of feeding - carbohydrates, proteins and lipids in the upper digestive tube, after the anatomical obstacle (stomach or jejunum) or at the gastric level in the cases with functional difficulties of swallowing.

The present study aimed to perform a medico-surgical comparative analysis of the 2 most widely used techniques: gastrostomy with peritoneal collar versus percutaneous endoscopic gastrostomy, based on the vast clinical experience in an Upper Digestive Surgery Clinic, with a permanent interest in surgical solutions of nutrition, initiated a long time ago by Prof. Gavriliu, MD, PhD, with a continuous updating to the recently developed minimally invasive approach.

## Materials and method

A retrospective study was carried out between January 2010 and January 2015 on the patients who underwent a surgical solution for feeding in our clinic. All the data were extracted from registered medical documents and operatory protocols. The indications, preoperative preparation, surgical techniques, and postoperative outcomes were analyzed.

## Results

Out of the 94 cases admitted for a surgical solution of feeding, 67 underwent gastrostomy with peritoneal collar (GPC) and in 27 cases, percutaneous endoscopic gastrostomy (PEG) was performed. 

The indications for GPC were benign or malign esophageal stenosis of several degrees, leading to the impossibility of a normal feeding and cachexia:

- post caustic esophageal stenosis (7 cases, 10.44%) 

- reposition of a previous gastrostomy (3 cases, 4.47%)

- incontinent gastrostomy (2 cases, 2.98%)

- advanced stages of malign tumors of tongue, pharynx, and larynx, the advancing of the endoscope to the esophageal tube was not technically possible (32 cases, 47.76%)

- mediastinal malignancies with mass effect or direct invasion of the esophagus (5 cases, 7.46%)

- advanced inoperable esophageal or eso-cardiac cancers (18 cases, 26,86%)

**Fig. 1:**
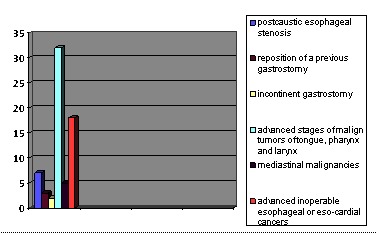
The indications for GPC

Patients who underwent PEG were suffering from neurological diseases or long-term comas treated in the intensive care unit:

- Parkinson disease (5 cases, 18.51%)

- advanced dementia (2 cases, 7.4%)

- sequelae of cerebral vascular accidents (12 cases, 44.44%), with absent gag reflex, depressed neurological state or severe lower cranial nerve palsies

- low Glasgow Coma Scale Score (8 cases, 29.62%) of different etiologies: severe craniocerebral trauma (3 cases), cirrhotic encephalopathy (1 case), neurological diseases (4 cases)

- early nasopharyngeal cancer (2 cases)

**Fig. 2:**
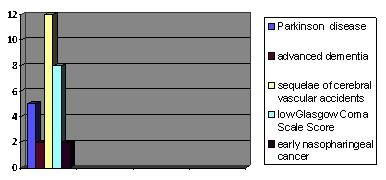
The indications for PEG

For all these cases, the written consent was obtained from the patients’ family or legal representative. Since almost all the patients were hospitalized for PEG placement, clearances from other services (cardiology, pulmonary, neurologic, etc.) were also obtained.

The preoperative preparation was according to the type of anesthesia.

For GPC, general anesthesia with orotracheal intubation was used in 47 cases (70.14%) and local anesthesia was reserved to cases with associated tracheostomy, severe cachexia or increased anesthetic risk. The surgical procedure was safe, well documented; the necessary instruments were simple and can be found in every surgical department. After a 4 cm length incision in the anterior abdominal wall, the stomach was identified under a direct visual control, a Pezzer tube was placed in the vertical part of the lesser gastric curvature, and a peritoneal collar was designed to assure its continence. Not more than 4 sutures are usually needed and the Pezzer tube was fixed with a nylon 5 suture to the skin and connected to a slope drainage system. The intraoperatory incidents were few and of minor importance. The integrity of the anatomical structure and vascularization of the stomach could be observed by tracking Gavriliu’s technique, with favorable influences on the future of the gastric function or repair.

The alimentation was initiated 48-72 hours post surgery, after testing gastric tolerance with glucose 5-10% solutions (500 ml/ 12 hours), first with liquids (milk), than with semisolid prepared meals. The approximate ratio per day, of 2000 calories included: 100 gx3 minced cooked meat, 3-4 eggs, 100 g sugar, 10 ml oil, 1-2 l milk [**1**]. The meals were initially frequent and in small quantity, of 50-100 ml, with progressive increase up to 200-250 ml during the next weeks, concomitant with a decrease in number/ day. The mean postoperative stay was of 3.4 days (3-5 days). The follow up period was of 30 days (2 controls/ week).

The postoperative evolution was good, with few complications:

- associated pyloric stenosis unrecognized preoperatory: reintervention was needed with pyloroplasty (1 case)

- local hemorrhage (2 cases, treated conservatory)

- transient gastric stasis > 1000ml/ 24 hours (7 cases)

- biliary reflux with associated gastric stasis ( 23 cases)

- Pezzer tube disinsertion (2 cases, chronic cougher patients, surgical repositioning was needed)

- ventilation problems with the blocking of tracheostomy with food and gastric material (5 cases, nursing of the 2 stomas).

For PEG, preoperative preparation was similar to the upper digestive endoscopy, namely 6-8 hours of fasting. The procedure might be safely performed even in patients with severe co-existing pathologies that associate an increased anesthetic risk.

The surgical technique implied the existence of a team, made up of a surgeon, an endoscopist, and an anesthetist. The surgeon visualized the light of the endoscope in the stomach by transillumination at the level of the anterior abdominal wall. The tube was placed percutaneous, by a 1cm length incision, with the help of the endoscopist who anchored the initial wire. It is an easy to learn technique, the equipment needed is expensive (the kit for PEG). We encountered some difficulties, especially in young patients, in the correct visualizing of the gastric vascular structure, needed for a correct placing of the feeding tube. 

**Fig. 3:**
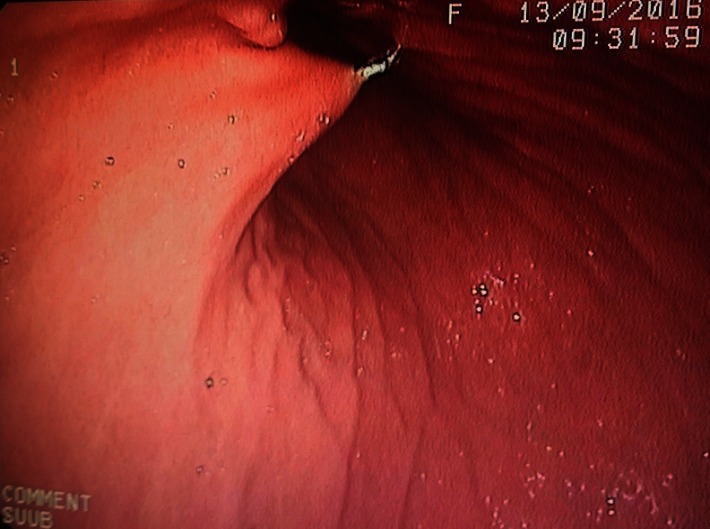
Needle and guide wire insertion under endoscopic control

**Fig. 4:**
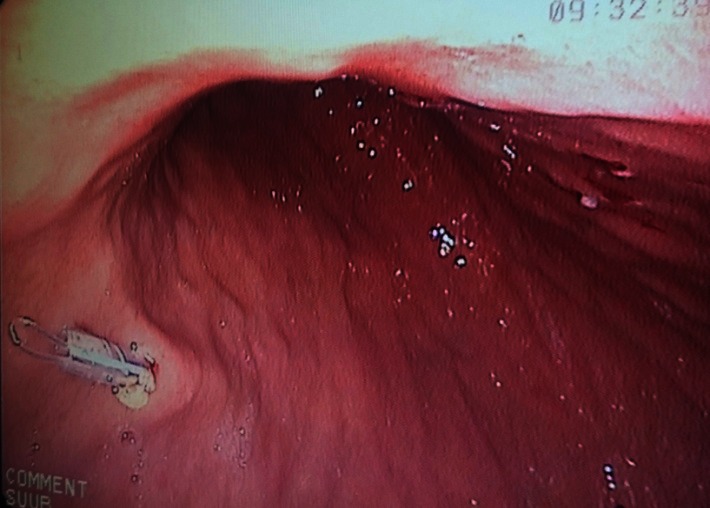
Gastrostomy tube in place

**Fig. 5:**
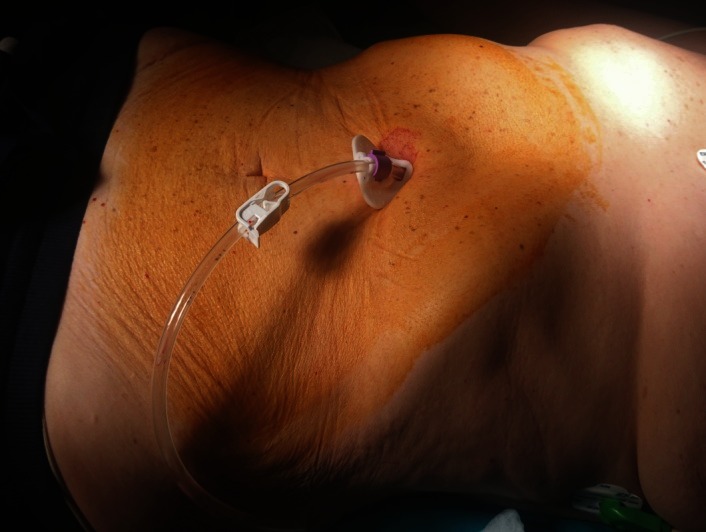
PEG – final aspect

The incidents were fewer than in classic GPC, with 1 case of biliary reflux and no cases of significant hemorrhage or gastric stasis > 1000 ml. The alimentation could be initiated immediately, but specifically designed prehydrolyzed nutritive solutions were needed, due to the small dimension of the feeding tube. This was a cost that had to be taken into account and assumed by the hospital and, on long term, by the patient’s family. Postoperatory evolution was more influenced by the underlying disease, than by the surgical procedure. 

## Discussions

The first major difference between the 2 techniques regards the indications, namely the correct choice of the underlying pathology that needs artificial feeding: if GPC addresses mainly to patients with anatomical obstruction (complete or incomplete stenosis) of the upper digestive tract, PEG is an option for the patients with functional swallowing disorders, the procedure needing the permeability of the upper digestive tract in order to pass through the endoscope.

A number of conditions compromise the passage of food along the digestive tract, but without affecting its intrinsic permeability, such as patients hospitalized in intensive care units or neurological patients [**2**,**3**]. Nasogastric tube (NGT) feeding is a classic, time-proven technique, although its prolonged use can lead to complications such as lesions to the nasal wing, chronic sinusitis, gastro-esophageal reflux, and aspiration pneumonia. Several studies showed that PEG is a safe and effective procedure, with fewer complications than NTG, that can be used when there is a need for enteral nutrition for a longer time period [**2**-**4**]. Several studies evidenced the role of PEG in nutrition of patients suffering from the neurological diseases such as acute stroke, respiratory impaired amyotrophic lateral sclerosis, advanced dementia and cerebral palsy [**5**-**9**].

On the other hand, distal enteral obstruction, severe uncorrectable coagulopathy, and hemodynamic instability constitute the main absolute contraindications for PEG tube placement in hospitalized patients [**10**,**11**].

Another indication for PEG is head and neck cancers, in patients with digestive tube still permeable for endoscopy [**12**-**14**]. We observed that the neoplastic patients wearing both tracheostomy and gastrostomy needed a complex nursing of the 2 tubes, the presence of alimentary material on the tracheostomy tube being frequently encountered. Another issue was the difficulty of oral communication with them, thus we consider that the postoperative nursing is more difficult than the surgical intervention itself. Due to the larger dimension of the tube, the aspiration risk and respiratory complications were more frequent when compared to PEG (5 vs. none). The clinical observation sustains that, in patients diagnosed with head and neck cancers that are inoperable and need tracheostomy, we recommend also the reevaluation of the digestive tube while it is still permeable, to perform a PEG and install a nutrition solution in “stand-by” before the evolution to dysphagia with a progressive degradation of the nutritional balance and cachexia. 

## Conclusions

The 2 surgical solutions of feeding complete one another and it is important to have the technical support and skills to perform both, attentively choosing the appropriate indication. GPC described by Gavriliu still remains an efficient surgical technique in the modern era, characterized by low-cost and wide addressability (it can be practiced in any surgical department with minimal instruments and even local anesthesia), addressing major feeding difficulties related to esophageal stenosis in various stages of therapy.

PEG may be an excellent minim invasive solution in cases with permeable digestive tube, but the costs and availability of the PEG kit and prehydrolyzed nutritive solution, as well as the co-existence of an upper digestive endoscopy service, are limitations that must be taken into account. 
